# Prevalence of myocardial crypts in a large retrospective cohort study by cardiovascular magnetic resonance

**DOI:** 10.1186/s12968-014-0066-0

**Published:** 2014-09-16

**Authors:** Nicholas Child, Tina Muhr, Eva Sammut, Darius Dabir, Eduardo Arroyo Ucar, Tootie Bueser, Jaswinder Gill, Gerry Carr-White, Eike Nagel, Valentina O Puntmann

**Affiliations:** Department of Cardiovascular Imaging, The Rayne Institute, Division of Imaging Sciences, King’s College London, 3rd Floor Lambeth Wing, St. Thomas’ Hospital Campus, London, SE1 7EH UK; Department of Cardiology, Guys and St Thomas’ Hospital, London, UK

**Keywords:** Myocardial crypts, Hypertrophic cardiomyopathy, Myocardial clefts, Left ventricular hypertrophy

## Abstract

**Background:**

Myocardial crypts are discrete clefts or fissures in otherwise compacted myocardium of the left ventricle (LV). Recent reports suggest a higher prevalence of crypts in patients with hypertrophic cardiomyopathy (HCM) and also within small samples of genotype positive but phenotype negative relatives. The presence of a crypt has been suggested to be a predictor of gene carrier status. However, the prevalence and clinical significance of crypts in the general population is unclear. We aimed to determine the prevalence of myocardial crypts in a large cohort of subjects using clinical cardiovascular magnetic resonance (CMR).

**Methods:**

Consecutive subjects referred for clinical CMR during a 12-month period (n = 1020, age 52.6 ± 17, males: 61%) were included. Crypts were defined as >50% invagination into normal myocardium and their overall prevalence, location and shape was investigated and compared between different patient groups.

**Results:**

The overall prevalence of crypts was 64/1020 (6.3%). In a predefined ‘normal’ control group the prevalence was lower (11/306, 3.6%, p = 0.031), but were equally prevalent in ischemic heart disease (12/236, 5.1%, p = n/s) and the combined non-ischemic cardiomyopathy (NICM) groups (24/373; 6.4%, p = n/s). Within the NICM group, crypts were significantly more common in HCM (9/76, 11.7%, p = 0.04) and hypertensive CM subjects (3/11, 27%, p = 0.03). In patients referred for CMR for family screening of inherited forms of CM, crypts were significantly more prevalent (10/41, 23%, p < 0.001), including a smaller group with a first degree relative with HCM (3/9, 33%, p = 0.01).

**Conclusion:**

Myocardial crypts are relatively common in the normal population, and increasingly common in HCM and hypertensive cardiomyopathy. Crypts are also more frequently seen in normal phenotype subjects referred because of a family history of an inherited cardiomyopathy and HCM specifically. It is uncertain what the significance of crypts are in this group, and because of variability in the imaging protocols used and their relative frequency within the normal population, should not be used to clinically stratify these patients. Prospective studies are required to confirm the clinical significance of myocardial crypts, as their significance remains unclear.

**Electronic supplementary material:**

The online version of this article (doi:10.1186/s12968-014-0066-0) contains supplementary material, which is available to authorized users.

## Background

Cardiovascular magnetic resonance (CMR) is recognized as the gold standard for in vivo determination and quantification of cardiac volumes, mass and dimensions [1–3], based on excellent spatial resolution and strong contrast between blood and myocardium. However, with increasingly advanced imaging the paradoxical dilemma of identifying novel findings with unknown clinical significance becomes increasingly problematic [4].

Myocardial crypts are slit-like blood-filled invaginations within the compacted LV myocardium, and have also previously been referred to as clefts and fissures. Although they were first identified macroscopically at autopsy in cases of HCM [5] they have subsequently been reported in a wide variety of cardiac diseases [6,7], as well as normal control subjects [8–10]. Microscopically described in the context of HCM, crypts are seen to extend inwards from the endocardial surface, lined by endocardium ending in a blind pocket [9] within areas of marked fascicular disarray [10]. Evidence of any clinical significance remains elusive and crypts are generally thought to represent a normal variant.

Recently however, myocardial crypts have been associated with HCM gene mutations. In a recent retrospective study (n = 300) patients with phenotypic HCM and a known disease-causing mutation were significantly more likely to have crypts, than in patients with phenotypic HCM without a known disease-causing mutation [11]. In patients without phenotypic HCM, but known to carry disease-causing mutations (‘carriers’) small studies have identified a high prevalence of myocardial crypts [8,12,13], suggesting a potential role of crypts to identify patients who should proceed to genetic testing.

There are a number of inconsistencies in the methodology of these studies, including the specific criteria for identifying crypts (whether the historical 50% penetration [8,12,14,15], or more recent 30% penetration [7] is used) and the need for additional non-standard views [7]. In the present study we undertook a systematic review to test the utility of various diagnostic criteria and to estimate the prevalence of crypts using CMR in a sufficiently large and relatively unbiased cohort.

## Methods

Consecutive subjects presenting during a 12-month period to the clinical CMR service of the Department of Cardiovascular Imaging at King’s College London were included encompassing a broad range of clinical backgrounds, from a mixed 2–3 million general population. Exclusion criteria for all subjects were the generally accepted contraindications to CMR (implantable devices, cerebral aneurysm clips, cochlear implants, severe claustrophobia) or history of renal disease with a current estimated glomerular filtration rate <30 mL/min/1.73 m^2^. The study protocol was reviewed and approved by an institutional ethics committee and written informed consent was obtained from all participants. Subjects with no evidence of heart disease on CMR (normal LV volumes, function and mass, and absence of Late Gadolinium Enhancement (LGE), who were normotensive, took no medication and had no family history of CM or sudden cardiac death served as a ‘control’ comparator group. Subjects who did not fulfill these criteria were divided into the following CMR groups; ischemic heart disease (IHD) with evidence of ischemic type scar on CMR and/or a positive stress test, NICM with evidence of increased left ventricular end-diastolic volume indexed to body surface area and reduced ejection fraction, compared with published reference ranges normalized for age and sex [14], and no ischemic scar or positive stress test, and a normal phenotype group who were referred for CMR screening due to a family history of an inherited CM (FHCM). This screening group consisted of patients with a first degree relative with a proven or suspected inherited CM (HCM, ARVC and DCM). The non-ischemic CM group was further sub-classified into dilated CM (DCM), hypertensive CM (HtCM), inflammatory CM (including myocarditis, sarcoidosis, systemic inflammatory conditions), HCM, arrhythmogenic right ventricular CM (ARVC), congenital heart disease, pericardial disease, and other (non ischemic CMP without possible further classification). Patients who did not fulfill any of the above criteria are grouped together in a general ‘other’ group (such as patients with coronary risk factors with normal CMR, or an unknown clinical background).

### Image acquisition

CMR studies were performed with the patient supine using a clinical 1.5 Tesla (T) or 3 T scanner (Philips Healthcare, Best, Holland) equipped with advanced cardiac package and a 32-channel coil. Standardized imaging protocols were performed in line with the clinical questions following the standardised protocols [15]. Standardized patient specific planning volumetric cavity assessment was obtained by whole-heart coverage of gapless short-axis slices (SAX). Thereafter, cine-images of 3 long-axis views (LAX: 4-chamber, 2-chamber and 3-chamber view) and transverse axial views were acquired. All cine-images were acquired using a balanced steady-state free precession (SSFP) sequence in combination with parallel imaging (SENSitivity Encoding, factor 2) and retrospective gating during a gentle expiratory breath-hold (TR/TE/flip-angle: 3.4 ms/1.7 ms/60°, spatial resolution 1.8×1.8×8 mm). LGE imaging was performed in corresponding views in all subjects using a mid-diastolic inversion prepared 2-dimensional gradient echo sequence (echo time/repetition time/flip angle 2.0 ms/3.4 ms/25°, spatial resolution 1.8 × 2 × 10 mm reconstructed to 1.8 × 1.8 × 8 mm, with a patient-adapted prepulse delay), 20 minutes after contrast injection (gadobutrol, 0.2 mmol/kg body weight). Imaging datasets with complete cine imaging and LGE imaging were included in the further analysis.

### Image analysis

All routine CMR analysis was performed using commercially available software (ViewForum, Extended Workspace, Philips Healthcare, Holland) and following societal post-processing guidelines [15–17]. Endocardial LV borders were manually traced at end-diastole and end-systole. The papillary muscles were included as part of the LV cavity volume. LV end-diastolic (EDV) and end-systolic (ESV) volumes were determined using Simpson’s rule. Ejection fraction (EF) was computed as EDV-ESV/EDV. LV mass was calculated as the difference between endocardial and epicardial contour area multiplied by the specific gravity of myocardium (1.05 g/mL). All volumetric indices were normalized to body surface area.

### Definition of crypts and analysis

Crypts were defined visually as a structural abnormalities consisting of narrow, deep blood-filled invaginations considered on cine viewing to penetrate >50% of the thickness of adjoining myocardium during diastole [9], perpendicular (45–135 degrees) to the endocardial border of otherwise normal compacted myocardium and evidence of subtotal or total obliteration during systole by surrounding tissue [7]. Because of the recent inconsistency in the classification of myocardial crypts as either 30 or 50%, we analyzed crypts > 50% in-line with the bulk of the available studies, but also recorded crypts in the 25-50% for further comparison. Their location was recorded based upon the 17-segment heart model recommended by the American Heart Association [17], and because recent studies have reported only inferior/ inferoseptal crypts [13], a pre-specified sub-analysis of this group was performed (defined as segments 3,4,9,10,15). The overall appearance of the crypts were subdivided into 3 groups; triangular (v shaped), a width of less than half the height (I shaped), or little difference in width and height (u shaped). Two independent observers confirmed the presence of all the crypts.

An assessment of inter-observer variability was performed using a random selection of 20-blinded cases. Concordance of crypts identification, as well as depth, position, shape and number were all assessed.

### Statistical analysis

All statistics were performed using SPSS Statistics, version 20 (IBM SPSS, New York, USA). Only crypts with >50% invagination into adjoining myocardium were considered in the main statistical analysis. Crypts with 25-50% invagination are reported and analysed additionally. Continuous data were compared using a student t test (or one-way ANOVA when comparing multiple groups). For categorical data a chi-squared test was performed, with a Fishers exact test when expected frequencies were less than 5. A Fliess’ Kappa calculation was used to test inter-observer variability. Data are presented as means with standard deviations (Mean ± SD) and a p-value of less than 0.05 was considered significant.

## Results

CMR studies of 1020 consecutive patients (age 52.6 ± 17,male 61%) were analysed and subject characteristics are presented in Table 1. The clinical backgrounds were available for 963 subjects. The most common referral group had a diagnosis of non-ischemic cardiomyopathy (37%), followed by patients who fulfilled our ‘control’ group criteria (30%), then ischemic cardiomyopathy (23%).

**Table 1:** **Baseline characteristics**

**n,%**	**Control 306 (30%)**	**ICM 236 (23%)**	**NICM 373 (37%)**	**Family screening 43 (4)**	**Other/unknown 62 (6)**	**p value**
**Age years (mean, range, SD)**	44.7, 17–82, 16.1	65.3, 30–90, 11.2	54.5, 16–87, 16.0	42.4, 21–75, 15.8	48.7, 22–83, 15.7	<0.01
**Gender (male)**	165 (53.9%)	179 (75.8%)	222 (59.6%)	20 (46.5%)	31 (50%)	<0.01
**EDV mL (SD)**	79.5 (14.4)	101.4 (39.0)	94.8 (34.8)	78.9 (14.4)	75.2 (13.7)	<0.01
**ESV mL (SD)**	31.8 (9.6)	51.5 (4.9)	52.4 (35.6)	32.4 (7.7)	31.1 (8.2)	<0.01
**EF% (SD)**	60.6 (6.0)	46.8 (16.6)	49.2 (17.5)	59.1 (6.2)	58.9 (7.3)	<0.01
**Mass g (SD)**	54.8 (17.2)	66.2 (22.2)	73.3 (24.3)	51.5 (14.2)	55.3 (17.5)	<0.01

ICM- ischemic cardiomyopathy, NICM- non-ischemic cardiomyopathy, EDV – end diastolic volume, ESV – end-systolic volume, EF – ejection fraction.

**Figure 1:**
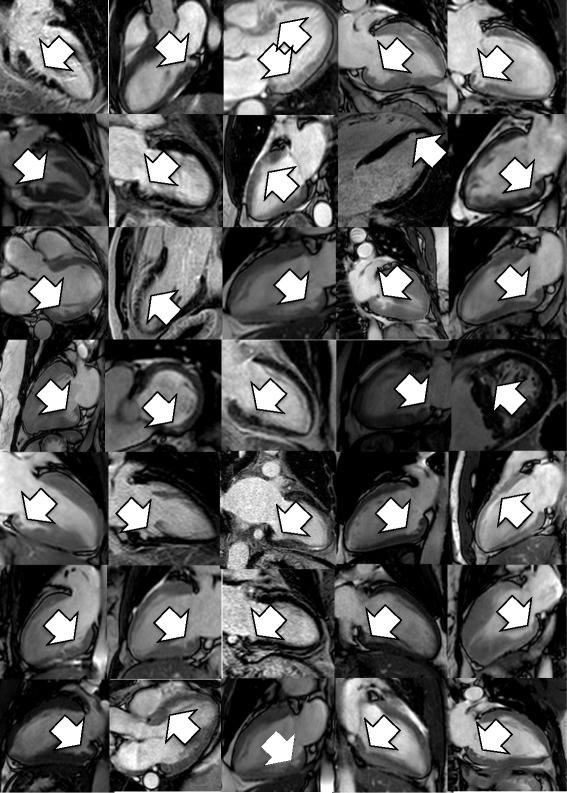
**Myocardial crypts idenitifed from 1020 consecutive CMR cases.** Examples of Myocardial crypts (>50% penetration) with white arrows indicating location.

Myocardial crypts (defined as >50% penetration) were identified in 64 out of 1020 patients (6.3%) (Table 2). The inter-observer agreement in identifying crypts that fulfilled the 50% criteria was very strong (K = 0.92). 62 of the crypts are presented in Figure 1.

**Table 2:** **Crypt prevalence**

	**Patients without crypts**	**Patients with crypts**	**Patients with partial crypts**
**Overall prevalence n- (%)**	927 (90.9%)	64(6.3%)	29 (2.8%)
**Age- years (SD)**	52.5 (17.0)	53.23 (16.2)	54.9 (16.7)
**Gender male/female (% male)**	565/362 (61%)	42/22 (66%)	15/14 (52%)
**Final Diagnosis- n (%)**			
ICM	214 (90.7%)	12 (5.1%)	10 (4.2%)
NICM	342 (91.0%)	24 (6.4%)	10 (2.7%)
DCM	120 (95.3%)	5 (3.9%)	1 (0.8%)
HtCM	8 (73%)	3 (27%)*	0
Inflammatory CM	79 (89.8%)	5 (5.7%)	4 (4.5%)
HCM	67 (87%)	9 (11.7)*	1 (1.3%)
ARVC	2 (50%)	0	2 (50%)
Congenital	4 (67%)	0	2 (33%)
Pericardial disease	7	0	
Other/	55	2	
Phenotype negative inherited cardiomyopathy family members (including HCM)	31 (72.1%)	10 (23.3%)**	2 (4.7%)
Phenotype negative HCM family members	6 (67%)	3 (33%)*	0
‘Control’ group	295 (96%)	11 (3.6%)*	
Unknown	45 (79%)	5 (8.8%)	7 (12.2%)

Distribution of Crypts by gender, age and clinical diagnosis. Crypts >50% invagination into surrounding normal myocardium. Partial crypts 25-50% invagination. ICM – ischemic cardiomyopathy, NICM – non-ischemic cardiomyopathy, DCM – dilated cardiomyopathy, HtCM – hypertensive cardiomyopathy, HCM – hypertrophic cardiomyopathy, ARVC – arrhythmogenic right ventricular cardiomyopathy. T-tests performed between crypts > 50% group and no crypt group. *p < 0.05, **p < 0.01.

**Figure 2:**
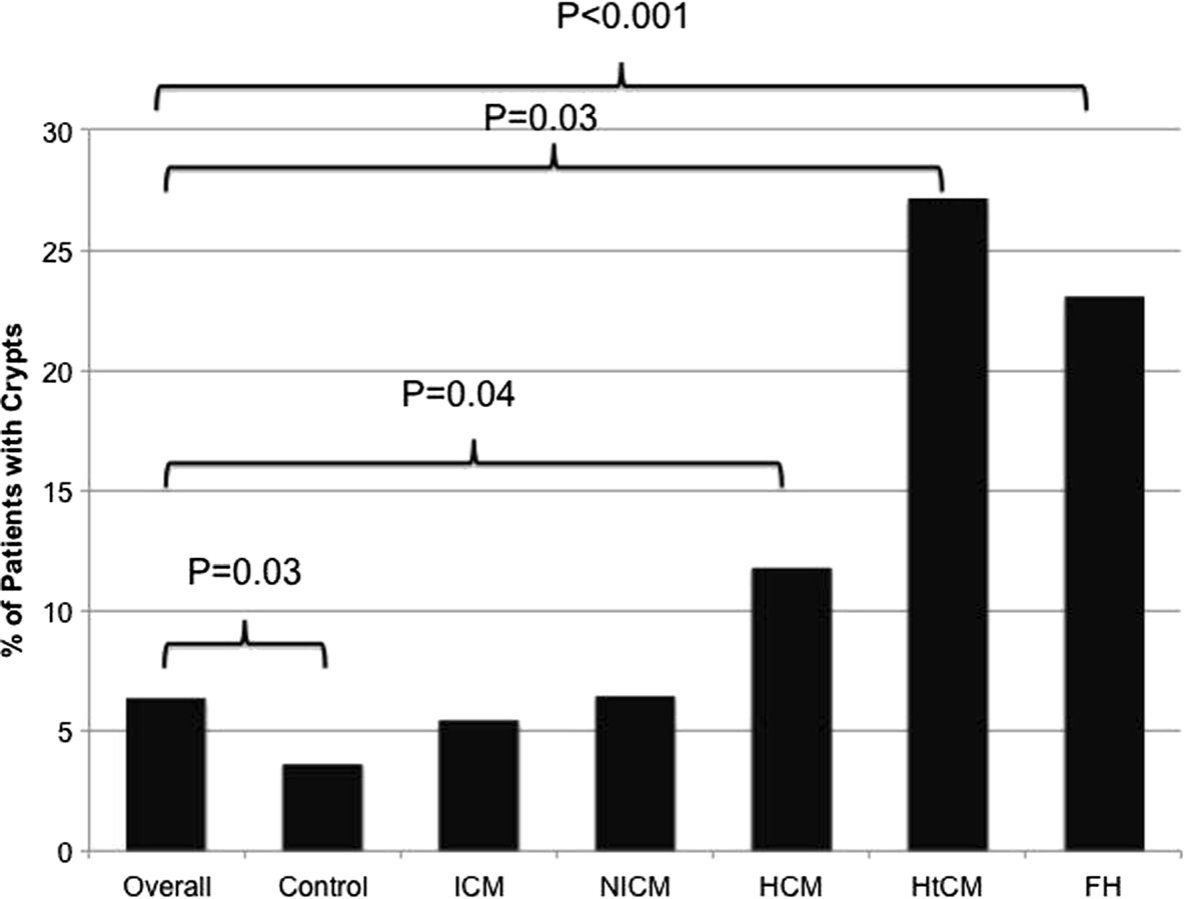
**Prevalence of myocardial crypts by underlying cardiac diagnosis.** ICM – ischemic cardiomyopathy, NICM – non-ischemic cardiomyopathy, HCM – hypertrophic cardiomyopathy, HtCM – hypertensive cardiomyopathy, FH – family history of cardiomyopathy.

Age and gender were not significantly different between the group with and without crypts. Myocardial crypts were found significantly less frequently in our ‘control’ group compared to the overall cohort (3.6% vs 6.3%, p < 0.031). They were found more frequently in the phenotypic HCM group (9/76, 12%, p < 0.044) and in the hypertensive CM group (3/11, 27%, p < 0.025), where they were all located in segment 4. The prevalence of crypts in the other NICM or ICM groups was not statistically different (Figure 2).

Crypts were also found more commonly in the phenotypic normal group referred for screening based on a FH of inherited cardiomyopathy (10/41, 23%, p < 0.001) compared to the overall group. Including the small subgroup of 9 patients referred with a 1^st^ degree relative with a clinical HCM diagnosis (3/9, 33%, p < 0.014) (Figure 2), again these were all located in segment 4 (AVI videos of these are included as on-line Additional files 1, 2, and 3). Of note, the only patient with a known disease-causing mutation did not have a myocardial crypt.

When crypts of >25% myocardial penetration are grouped together, the overall prevalence is 8.8% (90/1020), with the same trend between the diagnostic groups (less prevalent in the control group (3.6%, p < 0.001), more prevalent in the HCM group (13%, p = 0.1), HtCM (27, p = 0.68), FHCM (28%,p < 0.001) and the subgroup of the FHCM with HCM relatives (33%, p = 0.04). The smaller crypts were more difficult to differentiate from normal endocardial borders and papillary muscles, and there was less concordance between reviewers (K = 0.72).

Multiple crypts were found in 11 patients (1.1%). These were mainly in the NICM group (7/374, 1.8%, p = 0.1), but this was not statistically significant compared to the overall group. For the purpose of further analysis where more than 2 crypts were identified, only the most prominent 2 were analyzed. By far the most common location for myocardial crypts was in the basal inferior segments (segment 4 – 52/ 75 (69%), other inferior segments 9/75 (12%), non-inferior segments 14/75 (19%). U shaped crypts (36/75) and V shaped crypts (28/75) were the most common appearance (i shaped 11/75).

48 cases with inferior crypts (4.7%) were identified. Like the overall crypt group, inferior crypts were more common in the phenotypic normal group referred for screening for inherited cardiomyopathy (16%, p = 0.003), including the group referred for HCM screening (33%, p = 0.006). There was a non-significant trend for it to be more common in the overall NICM group (5.9%, p = 0.09), hypertensive CM group (27%, p = 0.012) and the HCM group (12%. P = 0.02), but less common in the ‘control’ group (2.6%, p = 0.03).

Myocardial crypts were most commonly identified from the LGE LAX view (64/75). The next best view was the pre-contrast cine LAX (60/75). When identifying a crypt using the LGE imaging it is very helpful to have a cine of the same crypt to differentiate blood pool invagination of the myocardium from scar.

### Volumes and systolic function

The mean EDV, ESV and SV were all better in the group with myocardial crypts compared to the group without (EDV 90.2 ± 31 vs 83.2 ± 25.0 mls, p = 0.09; ESV 45.9 ± 32 vs 36.5 ± 24 mls, p = 0.02; EF 58.2 ± 15 vs 58.2 ± 12%, p = 0.005. When subdivided by CM group, these differences were only apparent in the ICM group (EDV p = 0.053, ESV 0.02, EF 0.002).

## Discussion

To the best of our knowledge, this is the largest study to date to examine the prevalence of myocardial crypts using CMR within a broad clinical referral based population. We demonstrate that the overall prevalence of myocardial crypts is 6.3% in our cohort of over 1000 patients. We further show that although crypts are found less frequently in our control group, they are still present in 3.6% of apparently normal cases and they are by no means a rare phenomenon or specific for myocardial disease. Crypts were found significantly more frequently in patients with hypertensive cardiomyopathy and hypertrophic cardiomyopathy, in-keeping with previous studies [7,18,19]. Of particular interest, subjects who were phenotypic normal referred for CMR because of a family history of a presumed inherited CM had an increased prevalence of myocardial crypts, including a small subset with a 1^st^ degree relative with confirmed HCM.

**Figure 3:**
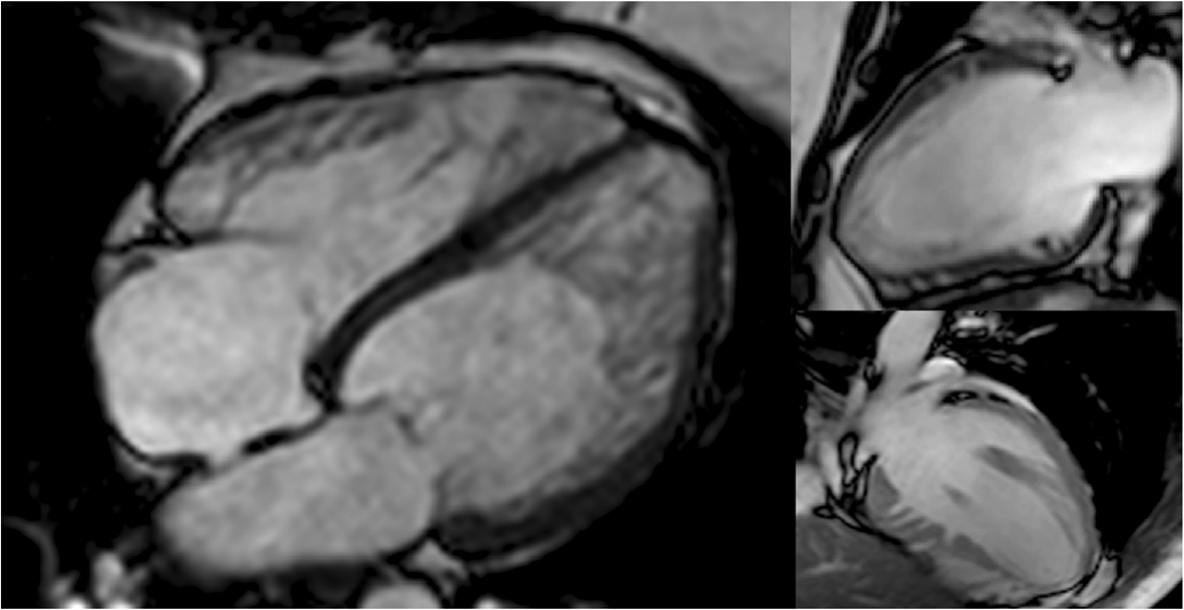
**Comparison between myocardial crypts and left ventricular non-compaction.** Left – A case of left ventricular non-compaction with “spongy” appearance of hyper-trabeculation overlying thinned compacted myocardium. Right (top and bottom) – prominent crypts seen to penetrate into normal myocardium.

Advances in cardiac imaging have led to novel findings and new diagnoses. However, identifying and differentiating new subtle pathological findings from physiological variation can be a challenge. Crypts were initially noted during post mortem analysis of patients with HCM [5], and represented a pathological manifestation with crypts occurring within areas of marked fascicular disarray [9,10]. Subsequently, with increasingly sophisticated invasive imaging techniques crypts have been identified in-vivo in multiple other cardiac pathologies as well as in normal hearts. The improved spatial resolution has improved our ability to differentiate deep crypts from certain disease states, such as left ventricular non-compaction (Figure 3), aneurysms and diverticulum [20]. However, it has also increased its recognition within the normal population, in where it is unlikely to be the manifestation of myocardial disarray but rather normal physiological variation.

Our overall prevalence of crypts was similar to other recent studies, reporting a prevalence of 6-7% in the general population and controls using the >50% criteria [8,12,13], and 12% when crypts >30% were included [7]. The increased prevalence of crypts in both hypertensive cardiomyopathy [12] and hypertrophic cardiomyopathy [13] is also in concordance with prior published reports. A recent study has identified crypts more commonly in HCM patients with a known disease-causing mutation on genetic testing, than those with normal genetic testing [11], possibly explaining the variability in reported HCM crypt prevalence seen in the literature. A previously reported increase seen in myocarditis was not apparent in our cohort [13].

The increased prevalence of myocardial crypts in patients referred because of a family history of a presumed inherited cardiomyopathy is a very interesting finding. There has been a very high proportion of crypts reported in subjects who are phenotypic normal with proven gene-causing mutations (‘carriers’). Recent studies by Germans et al. (n = 13, crypt prevalence 81%) [18], Brouwer et al. (n = 43, prevalence 70%) [7] and Maron et al. (n = 31, prevalence 61%) [19], have suggested that the presence of crypts in patients with an otherwise normal CMR and a family history of HCM could be a marker of ‘carrier’ status, and may have future potential to guide genetic testing. Our cohort of family screening referrals represented a relatively heterogeneous group in comparison, consisting of 9 patients referred with a first degree relative with a confirmed diagnosis of HCM (either genetically or clinically diagnosed), and 32 patients in whom there was a relative with either suspected HCM, or unexplained cardiac death in which an undiagnosed inherited cardiomyopathy was considered likely. Genetic testing was performed in only 5 of these relatives. This heterogeneity will likely explain why our prevalence of crypts was not as high as in these previous studies, it does however represent a general population referred for CMR in which there was an important but limited role in diagnosing HCM and screening of family members with HCM. Our study was designed to represent a real-world clinical referral group as opposed a tightly pre-selected research group, but supports the notion that whilst the presence of crypts is not entirely rare in the normal population there are certain clinical considerations within particular referral groups, albeit with caution.

An additional reason why our frequency of crypts in this group is not as high as in some reports is because of the use of additional imaging planes used in some studies. The vast majority of the crypts identified in this study were in the inferoseptal segments, particularly the basal inferior segments. The use of additional long-axis imaging through the inferoseptum is likely to further improve the identification of crypts in this area, with its use doubling the sensitivity of crypt detection in 1 study looking at HCM mutation carriers [7]. However, whilst it may be feasible to perform these extra views within prespecified groups, it is unlikely to be warranted in the majority of patients whilst the clinical significance of crypts remains unclear. Our findings support the notion that crypts are found more frequently in patients with a family history of HCM, and in these patients additional long-axis imaging may be warranted.

The improved spatial resolution has been credited with improving the ease of identifying crypts [19]. Traditionally crypts have been arbitrarily classified as >50% penetration into the myocardium [5], however a recent study has used an alternative 30% criteria based on the premise that this is considered abnormal to a substantial number of cardiologists [7]. Our clinical experience during this study was that we were a lot less confident in differentiating the smaller crypts (25-50%) from normal endocardial trabeculation, even though our intra-observer variability for this group remained relatively strong (K = 0.72). We would advocate that the 50% criteria are adopted in future studies for the purposes of consistency.

Furthermore, crypts were identified more frequently in patients with greater EF and smaller EDV and ESV independent of underlying cardiac diagnosis. This is similar to another recent study, which found lower EDV and greater stroke volume and ejection fraction in the group with myocardial crypts [13]. This phenomenon initially appears rather paradoxical, given the previous associations with myocardial disarray and disease. However, when we analyze each diagnostic group separately we find this difference only in the ischemic cardiomyopathy group. We hypothesize that the explanation for this finding is that in patients with thinned remodeled ischemic cardiomyopathy, which may be hypo- or akinetic the identification of myocardial crypts are more difficult. This mechanism would also suggest that crypts are easier to detect in subjects with LV hypertrophy, such as in patients with HCM and HtCM.

### Limitations

A few limitations apply to this study. Inclusion criteria relied on clinical referral in line with practice recommendations for clinical CMR; therefore, the inclusion of patients is not unselected and not necessarily representative of the general population. As such, although we are able to provide an insight into the prevalence of crypts in a large cohort with broad clinical questions, this may not be representative of the true normal population. However, our control group was also composed of a subgroup of normal healthy volunteers (n = 20) in whom the prevalence of crypts was similar (10%), and as such we believe that our control group remains representative. The greatest limitation is the absence of genome analysis available for our patients; hence we are unable to fully compare our findings with centers where genome analysis is routinely available. However, our study design reflects routine clinical practice and provides information on the prevalence of crypts in a less well defined more representative group of patients.

## Conclusion

Despite recent interest in the clinical significance of myocardial crypts, especially in the context of HCM mutation carriers, their clinical significance remains unknown. Crypts were found in 3.6% of our ‘normal’ subjects, and 6.3% overall. As such, they are a relatively common finding and are not specific for disease. They do however occur more frequently in certain diseases, such as HCM and hypertensive cardiomyopathy. In our study, they did occur more frequently in patients referred because of a family history of HCM and non-specific inherited cardiomyopathy with an otherwise normal CMR. This was a very broadly-selected real-world group and suggests that whilst the presence of crypts is not specific for HCM/ cardiomyopathy ‘carrier’ status, their appearance does have potential implications in such a pre-defined patient group. Future implications may include directing follow-up screening and genetic testing. However, prospective outcome data, both within the general population and HCM ‘carrier’ groups are required before clinical implications can be drawn. Because of the variability in sequences performed, and the subsequent variation in crypt prevalence within the HCM ‘carrier’ group we do not advocate using the presence of crypts to directly impact upon the choice of genetic screening on the current evidence.

## Additional files

Additional file 1
**Caption: Myocardial Crypt identified in patient with hypertrophic cardiomyopathy.**
Additional file 2
**Caption: Myocardial Crypt identified in patient with hypertrophic cardiomyopathy.**
Additional file 3
**Caption: Myocardial Crypt identified in patient with hypertrophic cardiomyopathy.**

## Abbreviation

CMR: Cardiovascular magnetic resonance
LV: Left ventricle
HCM: Hypertrophic cardiomyopathy
NICM: Non-ischemic cardiomyopathy
LGE: Late Gadolinium Enhancement
CM: Cardiomyopathy
IHD: Ischemic heart disease
FHCM: Family history of inherited cardiomyopathy
DCM: Dilated cardiomyopathy
HtCM: Hypertensive cardiomyopathy
ARVC: Arryhthmogenic right ventricular cardiomyopathy/ dysplasia
T: Tesla
SAX: Short-axis
LAX: Long axis
SSFP: Steady state free processing
EDV: End diastolic volume
ESV: End systolic volume
